# Emerging agents and regimens for multiple myeloma

**DOI:** 10.1186/s13045-020-00980-5

**Published:** 2020-11-09

**Authors:** Yang Yang, Yi Li, Huiyao Gu, Mengmeng Dong, Zhen Cai

**Affiliations:** 1 Bone Marrow Transplantation Center, Department of Hematology, The First Affiliated Hospital, College of Medicine, Zhejiang University, Hangzhou, Zhejiang China; 2 Institute of Hematology, Zhejiang University, Hangzhou, Zhejiang China; 3 Zhejiang Laboratory for Systems and Precision Medicine, Zhejiang University Medical Center, Hangzhou, Zhejiang China

**Keywords:** Multiple myeloma, Monoclonal antibody, Immunotherapy, Checkpoint inhibitor, BiTE, CAR-T

## Abstract

The outcomes of multiple myeloma (MM) have been improved significantly with the therapies incorporating proteasome inhibitors (PI), immunomodulatory drugs, monoclonal antibodies (MoAb) and stem cell transplantation. However, relapsed and refractory MM (RRMM) remains a major challenge. Novel agents and regimens are under active clinical development. These include new PIs such as ixazomib, marizomib, and oprozomib; new MoAbs such as isatuximab and MOR202; novel epigenetic agent ricolinostat and novel cytokines such as siltuximab. Recently, the first XPO-1 inhibitor, selinexor, was approved for RRMM. BCMA-targeted BiTE, antibody–drug conjugates and CAR-T cells have the potential to revolutionize the therapy for RRMM. In this review, we summarized the latest clinical development of these novel agents and regimens.

## Background

Novel agents and regimens as well as new technologies for tracing minimal residual diseases (MRD) have substantially improved the prognosis of patients with multiple myeloma (MM), with an increase in median survival from 3–5 years to 8–10 years in the past decade [1, 2]. The goal of treatment for newly diagnosed, transplant-eligible patients is to achieve the best depth of remission and improve the progression-free survival (PFS) and overall survival (OS).

The consensus is that induction therapy followed by autologous stem cell transplantation (ASCT) and maintenance therapy should be the overall management of myeloma, while the quality of life, tolerability, duration of treatment, convenience, and patients’ preference are taken into account [3]. For patients eligible for ASCT, three to four cycles of induction therapy are generally needed before the mobilization of hematopoietic stem cells. Proteasome inhibitors (PIs) and immunomodulator drugs (IMiDs)-based triplet regimens are preferentially considered, such as bortezomib (BTZ) and lenalidomide (Len) plus dexamethasone (DEX) (VRD), BTZ and thalidomide plus DEX (VTD), or cyclophosphamide and BTZ plus DEX (CyBorD). With better depth of remission and survival [4–6], VRD is currently a standard regimen. The quadruplet regimen of BTZ, Len, cyclophosphamide, and DEX has been found to have increased hematological toxicity but similar efficacy compared to the triplet regimens [7]. Furthermore, with the wide application of cytogenetics-based stratification standards, clinical trials are generally recommended to high-risk patients with the presence of del(17p), translocation t(4;14), t(14;16), t(14;20), 1q21 amplification or p53 mutation; or primary plasma cell leukemia; or the presence of 5 to 20% circulating plasma cells and extramedullary disease. Patients with more than one of the high-risk cytogenetic features are considered to have an “ultra-high-risk” disease. Based on the promising data from randomized trials [8, 9], the MAYO 2020 mSMART guideline recommended induction therapy with monoclonal antibody–based daratumumab (D)-VRD regimen for cytogenetically high-risk patients [10].

For patients ineligible for transplantation, the goal is to achieve deep remission without any serious adverse effects (AEs). Elderly patients who are often complicated with different comorbidities and various impairment of cognitive, physical, and social functions should be thoroughly evaluated before induction therapy. For fit patients, VRD is recommended for initial therapy followed by maintenance therapy with Len, or a BTZ-based regimen for high-risk patients. Daratumumab and Len plus DEX (DRD) is an alternative that has been recently approved for long-term treatment.

In the new drug era, the role of ASCT is still irreplaceable, particularly for patients with high-risk cytogenetic features [11]. ASCT could improve the depth of response, MRD-negativity rate, and PFS, but its benefit in OS needs further evaluation with longer observation time and more cases [12]. However, no consensus has been reached regarding the time for ASCT (early ASCT following induction therapy versus delayed ASCT after relapse) in patients with a standard-risk [12, 13]. Allogeneic hematopoietic stem cell transplant is rarely considered, but is applicable for young patients with high-risk cytogenetics. The significance of the second ASCT is unclear. Hence, more prospective, randomized trials are needed to clarify this in relapsed and refractory MM (RRMM).

Progression of disease is very common even when a complete remission (CR) has been achieved after ASCT. It is important to achieve MRD negativity through maintenance therapy to prolong the response duration and PFS without inducing severe AEs. Len is generally recommended for maintenance therapy [14, 15], and BTZ as well [16, 17], especially in patients with high-risks (e.g., del17p). Studies investigating the response to maintenance therapy with monoclonal antibodies are ongoing. Based on the benefit in PFS demonstrated in the TOURMALINE-MM3 (NCT02181413) study [18], ixazomib, a novel PI, has been included as a category A recommendation in NCCN guidelines for maintenance therapy in transplant-eligible patients with newly diagnosed MM (NDMM). Though the duration of maintenance therapy is still controversial, increasing evidence has suggested that it should be decided according to MRD status.

**Fig. 1:**
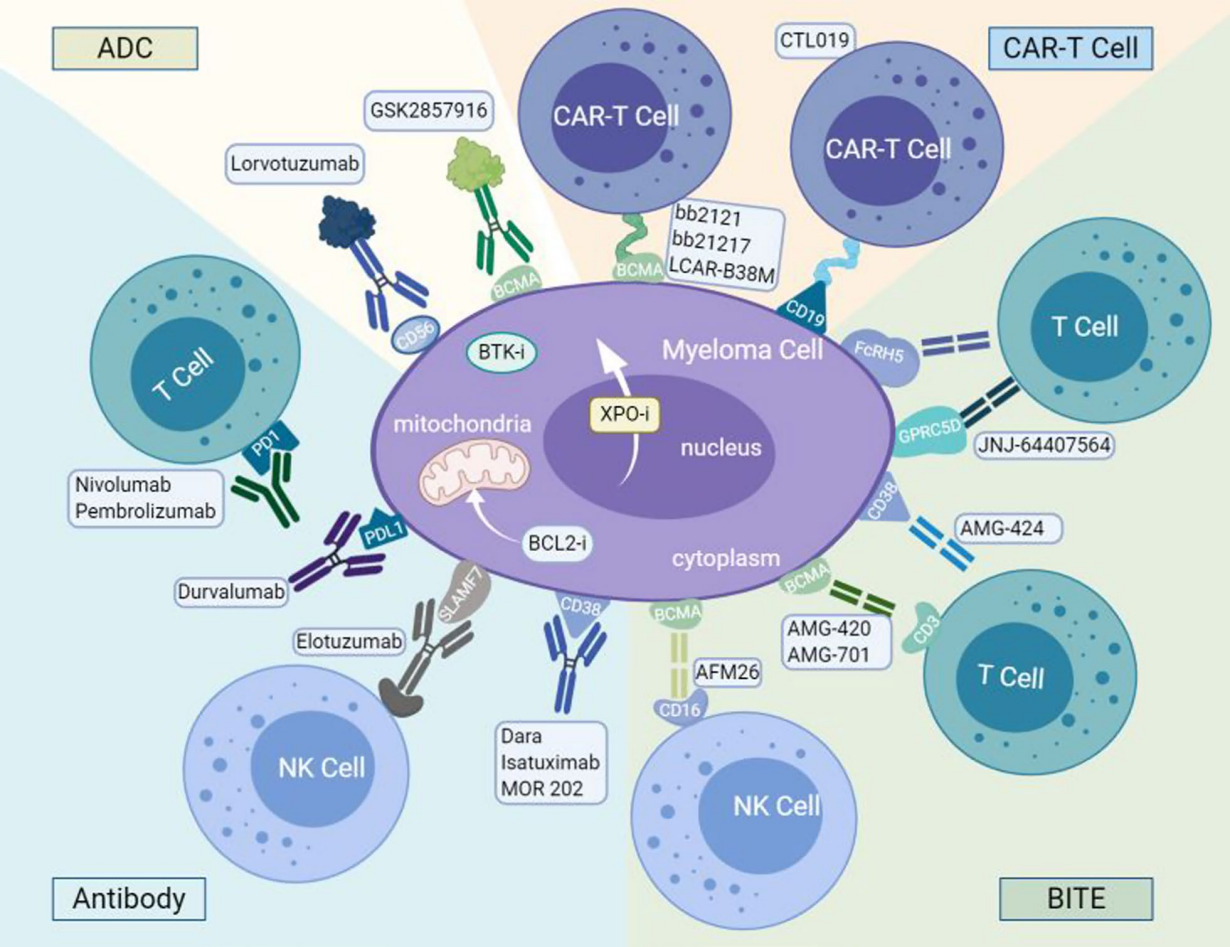
Therapeutic options for targeting myeloma

Currently, the treatment strategies for RRMM are based on the different combinations of conventional drugs and novel drugs, including monoclonal antibodies, PIs, IMiDs, alkylating agents, anthracyclines, and corticosteroids. It is suggested that a triplet regimen is better than a two-drug combination [19]. In addition, immune therapies, such as chimeric antigen receptor T cells (CAR-T), checkpoint inhibitors and vaccines, also play a promising role in the management of MM. Several different classes of these agents were discussed in this review, with a particular focus on the novel immunomodulatory agents. Figure 1 representatively summarizes selected agents, and Tables 1, 2, and 3 summarize key clinical trials.

**Table 1:** Clinical trials of non-immunotherapies

Agent		Frontline			Relapsed and refractory		
		Phase 1	Phase 2	Phase 3	Phase 1	Phase 2	Phase 3
Proteasome inhibitors	Carfilzomib (K)			ENDURANCE (NCT01863550): KRD versus VRD, similar PFS, 34.6 mon versus 34.4 mon, increased toxicity	NCT01998971: DKD (ORR 84%)	X-171-003-A1 (NCT00511238): single arm, ORR 23.7%, mOS 15.6 mon	1. ASIPRE (NCT01080391): KRD versus RD, better ORR (87.1 vs. 66.7%, *p* < 0.001), PFS (26.3 mon vs. 16.7 mon,* p* = 0.001) and 2-year OS (73.3% vs. 65.0%, *p* = 0.04)
							2. ENDEAVOR (NCT01568866): KD versus RD, better PFS (18.7 vs. 9.4 mon), ORR (77% vs. 63%), VGPR (54.3% vs. 26.8%) and CR (12.5% vs. 6.2%)
	Ixazomib			1. TOURMALINE-MM3 (NCT02181413): Ixazomib versus placebo, better median PFS (26.5 mon vs. 21.3 mon)			1. TOURMALINE-MM1 (NCT01564537): IRD versus RD, better PFS (20.6 mon vs. 14.7 mon), similar AEs
				2. TOURMALINE-MM4 (NCT02312258): Ixazomib versus placebo, better median PFS (17.4 mon vs. 9.4 mon), better TTP (17.8 mon vs. 9.6 mon), similar AEs (91% vs. 82%)			2. NCT01564537: IRD versus RD, better PFS (6.7 mon vs. 4.0 mon) and median OS (25.8 mon vs. 15.8 mon)
				3. TOURMALINE-MM2 (NCT01850524): IRD versus RD, ongoing			
				4. US MM-6: switching from BTZ-based to Ixazomib-based, similar ORR (62% vs. 65%), improved CR rate (4% to 22%)			
							2. OPTIMISMM(NCT 01,734,928): Pom + TZ + DEX versus BTZ + DEX, better PFS (11.2 mon vs. 7.1 mon)
							3. PomDe + CTX versus PomD: better ORR (64.7% vs. 38.9%)
Alkylating Agents	Bendamustine		Ben + BTZ + DEX (BBD): Overall response 91%, CR 9%	BenP versus MP: better CR (32% vs. 13%)	Ben + Ixazomib + DEX: VGPR 11%, PR 50%, mPFS 5.2 mon, OS 23.2 mon		
Bcl-2 inhibitors	Venetoclax				NCT01794507: Ven + BTZ + DEX: ORR 67%, VGPR 42%	1. STORM (NCT02336815): single arm, ORR 21%	1. BELLINI (NCT02755597): Ven + BTZ + DEX versus BTZ + DEX, suspended (safety issue)
						2. NCT02899052: Ven + KD, single arm, ORR 78%, VGPR 56%	2. CANOVA (NCT03539744): Ven + DEX versus Ven + Pom + DEX, ongoing
XPO-1 inhibitors	Selinexor				STOMP (NCT02343042): SVD, ORR 63%, median PFS 9 mon	STORM (NCT02336815): Sd, ≥ PR 26%, mDOR 4.4 mon, median PFS 3.7 mon, and mOS 8.6 mon	BOSTON (NCT03110562): SVD versus VD, better PFS (13.93 mon versus 9.46 mon), ORR (76.4% versus 26.3%)
Kinesin spindle protein inhibitors	Filanesib					(NCT00821249: Filanesib vs. Filanesib + DEX, similar PR rate (16% vs. 15%)	
	Melflufen					1. HORIZON (NCT02963493): single arm, ORR 30%, VGPR 11%, PR 18%, CBR 40%	
						2. O-12-M1 (NCT01897714): Melflufen + DEX, ORR 31%, CBR 49%

**Table 2:** Clinical trials of monoclonal antibodies, ADCs and BiTEs

Agent		Trial ID	Intervention	Disease type	Target	Efficacy data	Adverse event	References
Monoclonal antibodies	Daratumumab (Dara)	NCT01985126 SIRIUS	Single agent	RRMM	CD38	ORR 30.4%, mOS 20.5 mon	Grade 3 or 4 AEs:fatigue (3%), anemia (24%), thrombocytopenia (19%), neutropenia (12%), back pain (3%)	[42]
		NCT02136134 CASTOR	Dara + BTZ + DEX versus BTZ + DEX	RRMM	CD38	Better PFS (16.7 mon versus 7.1 mon, *p* < 0.0001), better ORR (83.8% vs. 63.2%, *p* < 0.0001)	Grade 3 or 4 AEs: thrombocytopenia (45.3% vs. 32.9%), anemia (14.4% vs. 16.0%), neutropenia (12.8% vs. 4.2%)	[43]
		NCT02076009 POLLUX	Dara + Len + DEX versus Len + DEX	RRMM	CD38	Better PFS (not reached versus 17.5 mon, *p* < 0.0001), and ORR (92.9% vs. 76.4%, *p* < 0.0001)	Grade 3 or 4 AEs: neutropenia (51.9% vs. 37%), anemia (12.4% vs. 19.6%), thrombocytopenia (12.7% vs. 13.5%), lymphopenia (5.3% vs. 3.6%)	[44]
		NCT03158688 CANDOR	Dara + Carfilzomib + DEX versus Carfilzomib + DEX	RRMM	CD38	Better ORR (84.3% vs. 74.7%, *p* = 0.004)	Grade 3 or 4 AEs: thrombocytopenia (24% vs. 16%), hypertension (18% vs. 13%), anemia (17% vs. 15%), pneumonia (13% vs. 9%), neutropenia (9% vs. 6%)	[46]
		NCT02195479 ALCYONE	Dara + Melphalan + Prednisone versus BTZ + Melphalan + Prednisone	NDMM	CD38	Better PFS (36.4 mon vs. 19.3 mon, *p* < 0.0001) and OS (78% vs. 67.9%)	Neutropenia (49.7% vs. 52.5%), thrombocytopenia (48.8% vs. 53.7%), peripheral sensory neuropathy (28.3% vs. 34.2%), anemia (28% vs. 37.6%)	[47]
		NCT02252172 MAIA	Dara + Len + DEX versus Len + DEX	NDMM	CD38	Better PFS (not reached vs. 33.8 mon, *p* < 0.0001)	Grade 3 or 4 AEs: neutropenia (50.0% vs. 35.3%), anemia (11.8% vs. 19.7%), lymphopenia (15.1% vs. 10.7%), pneumonia (13.7% vs. 7.9%)	[48]
		NCT02874742 GRIFFIN	Dara + Len + BTZ + DEX versus Len + BTZ + DEX	NDMM	CD38	Improved ORR (99% vs. 92%, *p* = 0.01), sCR (42.4% vs. 32%, *p* = 0.068), ≥ VGPR (91% vs. 73%, *p* = 0.001), ≥ CR (52% vs. 42%)	Grade 3 or 4 AEs: neutropenia (41.4% vs. 21.6%), lymphopenia (23.2% vs. 21.6%), thrombocytopenia (16.2% vs. 8.8%), leukopenia (16.2% vs. 6.9%), pneumonia (8.1% vs. 10.8%)	[49]
		NCT02541383 CASSIOPEA	Dara + BTZ + Thalidomide + DEX versus BTZ + Thalidomide + DEX	NDMM	CD38	Better sCR rate (29% versus 20%, *p* = 0.001), ≥ CR (39% vs. 26%, *p* < 0.0001), MRD negativity (64% vs. 44%, *p* < 0.0001), PFS rate of 18 mon (93% vs. 85%, *p* < 0.0001)	Grade 3 or 4 AEs: neutropenia (28% vs. 15%), lymphopenia (17% vs. 10%), stomatitis (13% vs. 16%)	[8]
	Elotuzumab (Elo)	NCT02654132 ELOQUENT-3	Elo + Pomalidomide + DEX versus Pomalidomide + DEX	RRMM	SLAMF7	Better PFS (10.3 mon vs. 4.7 mon), ORR (53% vs. 26%)	Grade 3 or 4 AEs: neutropenia (13% vs. 27%), anemia (10% vs. 20%), hyperglycemia (8% vs. 7%)	[53]
		NCT01478048	Elo + BTZ + DEX versus BTZ + DEX	RRMM	SLAMF7	Better PFS (9.7 mon vs. 6.9 mon), ORR (66% vs. 63%, ≥ VGPR (36% vs. 27%)	No additional clinically significant AEs occurred. Grade 1/2 infusion reaction rate was low	[54]
		NCT 02,272,803	Elo + Len + DEX versus Len + DEX	NDMM	SLAMF7	ORR (88% vs. 74%), ongoing	Grade 3 or 4 AEs: neutropenia (18% vs. 7%), leukopenia (15% vs. 0%). Any-grade infections (73% vs. 62%)	[55]
		NCT01668719 SWOGS1211	Len + BTZ + DEX with versus without Elo (maintenance therapy)	NDMM	SLAMF7	Similar PFS (31 mon vs. 34 mon, *p* = 0.449), ongoing	Fatigue (100%), peripheral sensory neuropathy (83%), edema (83%), lymphopenia (66%), leukopenia (50%)	[56, 57]
		NCT02495922 GMMG-HD6	Elo + BTZ + Len + DEX (induction therapy)	NDMM	SLAMF7	Similar ORR (82.4% vs. 85.6%, *p* = 0.35), similar ≥ VGPR (58.3% vs. 54.0%, *p* = *0*.*35)*	Ongoing	[58]
	Isatuximab (Isa)	NCT02990338 ICARIA-MM	Isa + Pomalidomide + DEX versus Pomalidomide + DEX	RRMM	CD38	Better PFS (11.53 mon vs. 6.47 mon, *p* = 0.001), ORR (60.4% vs. 35.3%), high-risk patients still had benefit	ALL grades: infusion reactions (56% vs. 0%), respiratory infection (43% vs. 26%), diarrhea (39% vs. 29%), similar anemia and thrombocytopenia; grade 3 or 4 AEs (87% vs. 71%)	[38, 61]
		NCT03275285 IKEMA	Isa + Carfilzomib + DEX versus Carfilzomib + DEX	RRMM	CD38	Better PFS (NR vs. 17.5 mon, *p* = 0.0007), ORR (86.6% vs. 82.9%), ≥ VGPR (72.6% vs. 56.1%, *p* = 0.193)	Grade 3 or 4 AEs: (76.8 vs. 67.2%)	[62]
	MOR202	NCT01421186	MOR202 monotherapy versus MOR202 + DEX versus MOR202 + DEX + Pomalidomide versus MOR202 + DEX + Len	RRMM	CD38	Ongoing, maximum tolerated dose was not reached	Grade 3 or 4 AEs: lymphopenia (38%), neutropenia (33%), leukopenia (30%)	[63]
Antibody−drug conjugates	GSK2857916	NCT03525678 DREAMM-2	Single agent	RRMM	BCMA	ORR 31% in the 2.5 mg/kg cohort and 34% in the 3.4 mg/kg cohort; mOS was not reached	Grade 3 or 4 AEs: keratopathy (27% in the 2.5 mg/kg cohort, 21% in the 3.4 mg/kg cohort), thrombocytopenia (20% and 33%), anemia (20% and 25%)	[68]
	Lorvotuzumab mertansine	NCT00346255	Single agent	RRMM	CD56	PR 5.7%; minor response 11.4%; SD 42.9%; mDOR 15.5 mon; PFS 6.5 mon	Grade 3 or 4 AEs: headache (5.4%), peripheral neuropathy (2.7%), neutropenia (2.7%)	[70]
BiTEs	AMG 420	NCT02514239	Single agent	RRMM	BCMA/CD3	sCR: 5; VGPR:1; PR:1; ORR 70%; DOR: 5.6–10.4 mon	Grade 3 or 4 AEs: infections (28%), polyneuropathy (4%)	[96]
	CC-93269	NCT03486067	Single agent	RRMM	BCMA/CD3	ORR 83.3%	Grade 3 or 4 AEs: neutropenia (52.6%), anemia (42.1%), infections (26.3%), thrombocytopenia (21.1%), 1 died of CRS	[97]

**Table 3:** BCMA-targeted CAR-T clinical trials

Trial ID	Conductor (reagent)	No. of evaluable patients	No. of median prior lines	Outcome	PFS	CRS	References
NCT02215967	National Cancer Institute	26	9.5	sCR: 2; CR: 1; VGPR:7; PR: 5; SD: 10; PD: 1; ORR 81%	EFS 31 weeks	2 G1, 7 G2, 3 G3, 1 G4	[94]
NCT02658929	Bluebird bb2121	33	7	sCR: 12; CR: 3; VGPR: 9; PR: 4; SD: 4; PD: 1; ORR 85%	11.8 months	23 G1–2, 2 G3	[96]
NCT03274219	Bluebird bb21217	22	7	ORR 83%; MRD negative: 10 at month 1	NA	5 G1, 7 G2, 1 G3	[97]
NCT03090659	Nanjing Legend LCAR-B38M	55	3	CR: 39; VGPR: 3; PR: 8; SD: 4; PD: 1; ORR 88%	20 months	46 G1–2, 4 G3	[98]
NCT03090659	Nanjing Legend LCAR-B38M	17	4	sCR: 13; VGPR: 2; SD:1; PD: 1; ORR 88.2%	12 months	10 G1–2, 6 G3, 1 G5	[100]
NCT03548207	Legend Biotech JNJ-68284528	29	5	sCR: 86%; PR: 3%; ORR 100%	26/29 (90%) PFS at median 9-mon follow-up	25 G 1–2, 1 G 3, 1 G5	[102]
NCT03430011	Bristol JCARH125	62	6	sCR + CR: 17; VGPR: 11; PR: 12; ORR 91%	NA	53 G1–2, 2 G3–4	[103]
NCT03915184	CARsgen Therapeutics CT053	13	4	CR: 2; VGPR: 6; PR: 4; ORR 100%	NA	1 G1, 1 G2, 1 G3	[104]
NA	Poseida Therapeutics MCARH171	11	6	ORR 64%; median DOR: 106 days	NA	4 G1–2, 2 G3	[105]
ChiCTR1800018137	Nanjing Iaso Biotherapeutics CT103A	9	4	CR: 4; PR: 3; MR: 1; SD: 1; ORR 100%	NA	Cases were G0–2	[106]

## Proteasome inhibitors

### Carfilzomib

The inhibitory effect of carfilzomib on proteasomes is stronger than that of BTZ. The open-label, single-arm phase 2 study PX-171-003-A1 (NCT00511238) [20] evaluated 266 patients with RRMM; 80% of the patients were refractory or intolerant to both BTZ and Len. The overall response rate (ORR) was 23.7% with median duration of response (mDOR) of 7.8 months. Median OS was 15.6 months. Common AEs were fatigue (49%), anemia (46%), nausea (45%), and thrombocytopenia (39%). Another study [21] analyzed the impact of cytogenetic abnormalities on the outcome of the PX-171-003-A1 study. The OS was comparable between patients with high-risk cytogenetics (del 17p13, t(4;14) or t(14;16) by interphase FISH or deletion 13 or hypodiploidy by metaphase cytogenetics) and patients with standard-risk group (25.8% vs. 24.6%; *p* = 0.85). A trend of shorter duration was observed in high-risk patients, including mDOR (5.6 vs. 8.3 months), PFS (3.5 vs. 4.6 months,* p* = 0.06) and OS (9.3 vs. 19.0 months, *p* = 0.0003). These results show that carfilzomib was at least partially efficacious in heavily pre-treated patients with high-risk cytogenetics.

In the ASPIRE (NCT01080391) study [22], compared with Len plus DEX (RD), addition of carfilzomib (KRD) achieved a higher rate of OS (87.1% vs. 66.7%, *p* < 0.001), a longer median PFS (26.3 vs. 17.6 months; *p* = 0.0001), and a better 2-year survival rate (73.3% vs. 65.0%; *p* = 0.04). A phase III head-to-head trial (ENDEAVOR; NCT01568866) directly compared the effect of carfilzomib and BTZ in patients with RRMM. All of the enrolled patients had been previously treated with one to three lines of therapy. The results showed that the KRD regimen was superior over the BTZ-based treatment in median PFS (18.7 vs. 9.4 months), ORR (77% vs. 63%), very good partial response (VGPR) (54.3% vs. 26.8%), CR (12.5% vs. 6.2%), and median survival time (47.6 vs. 40 months, *p* = 0.01 [23–25]. In a phase 1b study (NCT01998971) [26], daratumumab and carfilzomib plus DEX (DKD) were used in patients with RRMM who previously had one to three lines of therapy and were refractory to BTZ and Len. This trial demonstrated an ORR of 84% (79% in Len-refractory patients). Median PFS was not reached; 12-month PFS was 74% for all treated patients and 65% for Len-refractory patients. The incidence of drug-induced peripheral neuropathy was lower in the carfilzomib group than in the BTZ group. However, carfilzomib could be associated with severe adverse cardiovascular events, especially hypertension (mainly grade 1 or 2).

### Ixazomib

Ixazomib is the first oral PI approved by the FDA in November 2015. The phase III clinical trial TOURMALINE-MM1 (NCT01564537) [27] included 722 patients with RRMM who had been treated with one to three lines of therapies (including Len and PIs) and were randomized into the ixazomib and Len plus DEX (IRD) group or placebo plus RD group. The results showed that the median PFS of patients in the IRD group was significantly longer than that in the control group (20.6 months vs. 14.7 months, *p* = 0.01). The rates of serious AEs were similar in the two study groups (47% vs. 49%). A similar phase III clinical trial in China [28] showed that, compared to RD, IRD increased the median PFS (6.7 vs. 4.0 months, *p* = 0.035) and median OS (25.8 vs. 15.8 months; *p* = 0.001) in patients with RRMM.

Previous studies demonstrated that maintenance therapy with the IMiDs such as lenalidomide following ASCT could improve the survival and prolong the duration of disease control [15]. The phase III trial TOURMALINE-MM3 (NCT02181413) [18] investigated the efficacy of maintenance therapy with single agent ixazomib or placebo following ASCT. The trial showed that maintenance therapy with ixazomib (*n* = 395) increased median PFS to 26.5 months (95% CI 23.7–33.8) from 21.3 months (95% CI 0.58–0.89) in the placebo group (*n* = 261) (*p* = 0.0023), with a median follow-up of 31 months. The study also found that ixazomib increased MRD negativity (12% vs. 7%) and reduced the risk of disease progression or death by 28% compared to placebo. The updated data of the TOURMALINE-MM4 (NCT02312258) trial [29] evaluated the efficacy and safety of ixazomib vs. placebo as post-induction maintenance in ASCT-ineligible patients with NDMM. Patients who had received induction therapy and achieved at least a PR were randomized to the ixazomib or placebo group. The ixazomib group showed a significant improvement in median PFS vs. compared to the placebo group (17.4 vs. 9.4 months, 95% CI 0.542–0.801, *p* < 0.001). A significant benefit in PFS was seen in patients who achieved CR/VGPR during post-induction treatment (median 25.6 vs. 12.9 months, HR 0.586). Time to progression (TTP) was significantly prolonged in patients treated with ixazomib (median 17.8 vs. 9.6 months, HR 0.655, *p* < 0.001). OS data had not been reached (study was ongoing). The AEs were similar (91% vs. 82%) in the 2 groups and were mostly grade 1–2 (37% vs. 23% for grade ≥ 3 treatment emergent AEs).

Recently, a multicenter phase II study compared the efficacy of ixazomib and Len, both being the grade A recommendation for the maintenance therapy of standard-risk patients after ASCT and consolidation therapy with IRD [30]. The results showed that the rate of ≥ VGPR was similar between the two groups after a 1-year follow-up (ixazomib 80% vs. Len 88%) and both were well tolerated. However, the proportion of patients who discontinued the treatment due to disease progression was higher in the ixazomib group than that in the Len group.

Due to the convenience of oral administration and high safety requirements of the injection agents, a US community-based phase 4 study (US MM-6) [31] assessed the response of newly diagnosed, transplant-ineligible patients after switching from an injection PI (BTZ) to an oral PI (ixazomib). After three cycles of BTZ-based combination therapy, patients with a response of ≥ stable disease (SD) were changed to the oral administration of IRD. While the overall response rate did not change significantly (62% vs. 65%), the CR rate increased from 4 to 22% after the conversion. The records demonstrated good drug compliance, indicating that the oral regimen could be an option for home care unit program.

## IMiDs

### Pomalidomide

POM is one of the second-generation IMiDs. Previous studies demonstrated that POM could not only directly inhibit the proliferation of MM cells and suppress angiogenesis but also exert immunomodulatory effect on bone marrow microenvironment [32]. In 2013, POM was approved by the FDA for refractory patients who had been treated with at least two strategies (including BTZ and Len) earlier [33]. The phase III clinical trial MM-003 (NCT01311687) [34] compared the effect of POM and low-dose DEX (LoDEX) with POM and high-dose DEX in 455 patients with RRMM who had not responded well to BTZ and Len. The median PFS (4.0 vs. 1.9 months), median OS (13.1 vs. 8.1 months), and the ORR (≥ PR; 32% vs. 11%) were all better in the POM and LoDEX group than in the POM and high-dose DEX group.

In the phase III OPTIMISMM (NCT01734928) study, 559 patients resistant to Len were randomly assigned to POM and BTZ (Vel) plus DEX (PVD) group (281 patients) and the BTZ and DEX (VD) group (278 patients). The results showed that PFS significantly improved in the PVD group compared with the VD group (median 11.20 months vs. 7.10 months; *p* < 0.0001) [35]. Other triplet options, such as POM and DEX (PomD) plus cyclophosphamide, could significantly increase the ORR, compared with the PomD regimen in Len resistant patients with RRMM (64.7% vs. 38.9%; *p* = 0.0355) [36]. Some other studies also demonstrated that combination of POM or carfilzomib with bendamustine and DEX achieved good responses (ORR 61%) in patients with RRMM [37], with median PFS of 9.6 months (95% confidence interval (CI) 6.8–18.0) and median OS of 21.3 months (95% CI 12.3–N/A). The effects of the combination of monoclonal antibodies with POM were also investigated in several ongoing studies (such as APOLLO and ICARIA-MM) [38, 39], which offered alternatives for patients with RRMM. Safety analysis by a 5-year follow-up in the MM-010 study [40] showed that the most severe grade 3 or 4 hematological AEs were neutropenia (49.7%), followed by anemia (33%) and thrombocytopenia (24.1%), which were associated with the dose of POM and the number of treatment cycles.

## Monoclonal antibodies

### Daratumumab

Daratumumab is the first immunotherapeutic monoclonal antibody against CD38. After a follow-up of 3 years, the pooled analysis of the GEN501 and SIRIUS studies showed that monotherapy with daratumumab achieved the median OS of 20.5 months and ORR 30.4% in patients with RRMM [41, 42]. The CASTOR (NCT02136134) trial investigated the efficacy of daratumumab combined with BTZ and DEX (DVD) in patients with RRMM. After a median follow-up of 19.4 months, the results showed that, compared with the VD group, DVD regimen prolonged median PFS (16.7 vs. 7.1 months, *p* < 0.0001) and improved the ORR (83.8% vs. 63.2%, *p* < 0.0001) [43].

The phase III POLLUX study (NCT02076009) [44] showed that DRD treatment in patients with RRMM significantly increased ORR compared with RD treatment (93% vs. 76%; *p* < 0.001), while the median PFS was not reached. Subsequently, this study extended the follow-up time by 1 year with a median follow-up of 25.4 months [45]. The PFS in the RD group was 17.5 months, while the PFS in the DRD group had not been reached yet (*p* < 0.0001). The ORR was 92.9% vs. 76.4% and CR or better was 51.2% vs. 21.0% in the DRD and RD groups (both *p* < 0.0001). Subgroup analysis demonstrated that the PFS was better in the DRD group, irrespective of how many lines of treatment patients had received previously.

Benefit in PFS was also observed when daratumumab was added to carfilzomib and DEX (KdD versus Kd) for the patients with RRMM in the phase III CANDOR study (NCT03158688) [46]. The ORR was 84.3% and 74.7% (*p* = 0.0040) for KdD and Kd, respectively, with a median follow-up of 16.9 months.

A series of clinical trials were conducted to investigate the efficacy of daratumumab in patients with NDMM as a front line therapy. The ALCYONE (NCT02195479) study compared the effect of BTZ, melphalan, and prednisone (VMP) with VMP plus daratumumab (D-VMP) in patients with NDMM who were ineligible for HSCT. After a median follow-up of 40 months, the median PFS in D-VMP group is 36.4 months, which was significantly longer than that in VMP group (19.3 months; *p* < 0.0001). Patients in D-VMP group also had a benefit in OS (78% vs. 67.9%) [47].

The MAIA (NCT02252172) study in Europe [48] evaluated the efficacy and safety of DRD versus RD regimen in transplant ineligible patients with NDMM. After a median follow-up of 28 months, the DRD regimen showed an increase in the median PFS (not reached vs. 33.8 months, *P* < 0.0001) and reduced the risk of disease progression or death by 44%. Patients in the DRD group had at least a 20% improvement in stringent complete response (sCR) and more than a threefold increase in the MRD-negativity rate. These results support the use of daratumumab-based combination regimens as a first-line therapy for patients with NDMM.

The phase II ongoing GRIFFIN study (NCT02874742) [9] explored the effect of daratumumab combined with the VRD (D-VRD) in 207 ASCT-eligible patients with NDMM and evaluated the efficacy of daratumumab in maintenance therapy. Patients were treated with four cycles of induction chemotherapy (D-VRD), then received ASCT and two cycles of consolidation therapy, followed by 24-month maintenance therapy with Len alone or in combination with daratumumab. The study found that the sCR rate was higher in D-VRD group than in VRD group (42.4% vs. 32.0%; *p* = 0.1359) at the completion of consolidation therapy. In addition, the ORR (99% vs. 92%), ≥ VGPR (91% vs. 73%), and ≥ CR (52% vs. 42%) were all significantly improved in the D-VRD group compared with the VRD group. However, grade 3 or 4 hematological toxicity was increased in the D-VRD group.

The European trial CASSIOPEA (NCT02541383) [8] assessed the efficacy of daratumumab when added to VTD (D-VTD group, *n* = 543; VTD group, *n* = 543) during induction and after ASCT in patients with NDMM who were transplant-eligible. In the induction phase, daratumumab-based regimen was associated with a 53% reduction in the risk of progression or death. One hundred days after transplantation, 29% of patients in the D-VTD group and 20% of patients in the VTD group had achieved a sCR (*p* = 0.001). MRD-negativity by flow cytometry was 64% (346 out of 543) in the D-VTD group and 44% (236 out of 542) in the VTD group (*p* < 0.0001). After a follow-up of 18 months, the PFS rate in the D-VTD group was significantly higher than that in the VTD group (93% vs. 85%; *p* < 0.0001). Moreover, D-VTD also improved the remission depth for patients with high-risk cytogenetics (hazard ratio HR = 0.67) and in International Staging System stage III (HR = 0.66). Maintenance therapy of this trial is still ongoing. For the first time, these findings showed that addition of daratumumab improved the depth of response and PFS with acceptable safety in transplant-eligible patients with NDMM.

It is worth noting that daratumumab-based combinations could improve PFS regardless of the cytogenetic risk status for RRMM in CASTOR and POLLUX studies. In MAIA study, benefit of adding daratumumab was more significant in the standard-risk patients than in the high-risk patients with NDMM. However, the association between survival and cytogenetic abnormalities was not analyzed in these studies, mainly due to the small sample size in subgroups.

### Elotuzumab

Elotuzumab is a SLAMF7-targeting monoclonal antibody that could attack MM cells through NK cell–mediated antibody-dependent cytotoxicity and direct activation of NK cells. Elotuzumab monotherapy has not been shown to be beneficial, but a series of studies demonstrated its effect in combination with iMIDs for RRMM. A phase III clinical trial (ELOQUENT-2; NCT01239797) compared the efficacy of elotuzumab plus RD (ERD) with that of RD in patients with RRMM. The results showed that the PFS and ORR in the ERD group were 19.4 months and 79%, respectively, which were significantly better than those in the RD group (14.9 months, 66%). Such advantages of ERD were also found in the subgroup analysis. In addition, the elotuzumab group also showed improved OS compared with the RD group (43.7 vs. 39.6 months) with an extension of follow-up time [49–51]. Based on the findings in the ELOQUENT-2 study, the ERD regimen was approved by the FDA in November 2015 for patients who had been treated with one to three strategies earlier.

Based on the results of ELOQUENT-2, the phase II ELOQUENT-3 (NCT02654132) [52] investigated the addition of elotuzumab to pomalidomide plus DEX (E-Pd vs. Pd). In this trial, 117 patients with RRMM randomly received either E-Pd (*n* = 60) or Pd (*n* = 57). After a follow-up time of 9.1 months, the median PFS was 10.3 months in the E-Pd group and 4.7 months in the Pd group (*p* = 0.008), and ORR was 53% and 26%, respectively (odds ratio 3.25; 95% CI 1.49–7.11). Unfortunately, the trial was discontinuated as a result of disease progression (43% in the E-Pd group and 56% in the Pd group).

A phase II clinical trial (NCT01478048) showed that, compared with bortezomib and DEX (BD), BD plus elotuzumab significantly increased the median PFS (9.7 vs. 6.9 months) in patients with RRMM [53]. Another phase II study (NCT 02,272,803) [54] evaluated the efficacy and safety of elotuzumab plus RD (ERD) versus RD in newly diagnosed, transplant-ineligible patients in Japan. Latest results showed that ORR was 88% [70% (CI) 80–93] in the ERD arm (the PFS data was not reached).

The phase I/II study SWOGS1211 (NCT01668719) was designed to investigate whether deep remission early during induction therapy could be achieved in high-risk patients. This was the first trial to evaluate the effectiveness and safety of 8 cycles of VRD as induction and maintenance therapy with/without elotuzumab in newly diagnosed high-risk patients. Patients were defined as high risk if they had high-risk gene expression profile, such as t(14;16), t(14;20), del (17p) or 1q21 amplification, primary plasma cell leukemia or increased level of LDH. In the phase 1 of the trial, six patients completed eight cycles of induction therapy and five patients completed four cycles of maintenance therapy, both with elotuzumab plus VRD (Elo-VRD) with doses adjusted. The results showed that elotuzumab did not introduce additional treatment-related toxicities [55]. The phase 2 of the trial was ongoing, and the results reported at the European Hematology Association (EHA) 2020 [56] showed that, with a median follow-up of 53 months, the PFS in Elo-VRD group was not significantly different from that in VRD group (31 months vs. 34 months, *p* = 0.449). OS was 68 months in patients who had received Elo-VRD treatment but was not reached in VRD group. 72% of the 103 patients enrolled had grade ≥ 3 AEs.

Similarly, an ongoing phase III GMMG-HD6 trial (NCT02495922) aimed to investigate whether the addition of elotuzumab to VRD during induction, consolidation and maintenance could improve PFS in transplant-eligible patients as frontline therapy. Date published at EHA 2020 annual meeting [57] showed that ORR in Elo-VRD and VRD group was 82.4% and 85.6% (*p* = *0*.*35*), respectively, after 4 cycles of induction therapy. The rate of VGPR or better (≥ VGPR) was 58.3% and 54.0% in the Elo-VRD vs. and VRD groups (*p* = 0.35), respectively. These results showed that the addition of elotuzumab to VRD in newly diagnosed, transplant-eligible myeloma patients did not improve ≥ VGPR after induction therapy. Dimopoulos et al. [52] compared the elotuzumab and PomD regimen versus PomD regimen in patients with RRMM and showed that the ORR (53% vs. 26%) and PFS (10.3 vs. 4.7 months) were significantly improved by the three-drug regimen. In November 2018, elotuzumab, in combination with POM and LoDEX, was approved for the treatment of patients who had been treated earlier with at least two regimens (including Len and a PI).

### Isatuximab (SAR-650984)

Isatuximab is a chimeric immunoglobulin G1 (IgG1) monoclonal antibody that targets a specific epitope on CD38 and directly induces programmed cell death. In a phase I dose-escalation trial, isatuximab monotherapy was applied to patients with RRMM with the ORR and CR of 24% and 6%, respectively. Subgroup analysis showed that in the high-dose group, the ORR and CR were increased to 33% and 11%, whereas the toxicity did not differ significantly [58]. Another clinical trial [59] evaluated the effect of isatuximab plus RD in patients with RRMM who had received more than 2 lines of chemotherapy with 52% of them resistant to Len. The results showed that the ORR was 56% and the median PFS was 8.5 months after the triplet treatment [59].

A phase III clinical trial (ICARIA-MM; NCT02990338) [60] assessed the effect of isatuximab plus PomD (IPD) versus PomD in patients with RRMM resistant to BTZ and/or Len. All these patients had received more than two lines of previous therapies. The findings demonstrated that, compared with PomD, IPomD with low-dose DEX reduced the risk of disease progression and death by 40% (*p* = 0.001). The median PFS in the isatuximab group was 11.53 months, which was almost twofold of that in the control group (6.47 months). The ORR in the isatuximab group also increased significantly (60.4% vs. 35.3%). This survival benefit was observed in all the subgroups, including the subgroup with patients ≥ 75 years old, the subgroup with renal dysfunction, and the subgroup with Len-refractory disease. In addition, for high-risk patients with del(17p), t(4;14), and/or t(14;16), the ORR in the IPomD group was 50%, as compared with 16.7% in the PomD group. The IPomD group showed a median PFS of 7.5 months in high-risk patients, as compared with 3.7 months in the PomD group, and the risk ratio was 0.66 (0.33–1.28) [38].

IKEMA (NCT03275285) [61], a phase III trial, evaluated the efficacy of the combination of isatuximab with carfilzomib plus DEX (Isa-KD) in patients with RRMM who had received 1–3 lines of therapy previously. Of the 302 patients enrolled, 90% and 78% had been exposed to BTZ and Len, respectively, and 24% had high-risk cytogenetic abnormalities. With a median follow-up of 20.7 months, PFS in the Isa-KD group was not reached as compared with 17.5 months in the KD group (HR 0.531, 99% CI, 0.318–0.889, single arm, *p* = 0.0007). The ORR was 86.6% and ≥ VGPR was 72.6% in the Isa-KD group, compared with 82.9% and 56.1%, respectively, in the KD group (single arm, *p* = 0.1930). AEs (≥ grade 3) were 76.8% in the Isa-KD group and 67.2% in the KD group. The Isa-KD regimen was well tolerated in the trial.

#### MOR202 (TJ202)

MOR202 is another IgGl monoclonal antibody that targets human CD38. A multicenter, open-label phase I/II trial in Germany and Austria on patients with RRMM explored the safety of MOR202 (NCT01421186). A total of 91 patients were enrolled, including 35 in the MOR202 monotherapy group and 56 in the MOR202 combination therapy group (18 with MOR202 plus DEX, 21 with MOR202 plus DEX and POM, and 17 with MOR202 plus RD). The regimen was intravenous infusion of MOR202 for 30 min with DEX (40 mg), or in combination with DEX plus Len (25 mg) or POM (4 mg). The most common grade ≥ 3 AEs were lymphocytosis in 35 cases (38%), neutropenia in 30 cases (33%), and leukopenia in 27 cases (30%) [62]. The phase III trial of MOR202 to verify its efficacy in combination with RD in patients with RRMM is ongoing.

## Antibody–drug conjugates

Antibody–drug conjugates (ADCs), which are composed of a mAb linked to a cytotoxic drug via a biodegradable linker, are among the fastest growing anticancer drugs. A novel promising ADC for MM in clinic is the B-cell maturation antigen (BCMA)-targeted antibody, GSK2857916 (belantamab mafodotin), which is formed by a humanized IgG1 conjugated with a tubulin polymerization inhibitor, monomethyl auristatin F (MMAF). BCMA is a member of the TNF-receptor superfamily expressed only in the plasma cells and mature B lymphocytes [63]. Upon binding to its proliferation-inducing ligand, BCMA promotes MM cell growth and drug resistance [64]. The expression of BCMA in MM cells increases as disease progresses, rendering it an ideal antigenic target.

The multicenter, open-label, phase I study GSK2857916 (DREAMM-1, NCT02064387) enrolled 38 patients in dose escalation phase (part 1) and 35 patients in dose expansion phase (part 2). All patients had been diagnosed with RRMM [65]. In part 1, patients received GSK2857916 (0.03–4.60 mg/kg) intravenously once every 3 weeks for up to 16 cycles. In part 2, patients received the recommended dose of 3.40 mg/kg once every 3 weeks. There was no dose-limiting toxicity in part 1. Corneal symptoms caused by MMAF toxin were common (53% in part 1 and 63% in part 2), including blurred vision, dry eye, and photophobia, all of which were reversible. Two patients discontinued treatment in part 1 because of the corneal events and none discontinued in part 2. The most common grade 3 or 4 toxicities were thrombocytopenia (34% in both part 1 and part 2) and anemia (16% in part 1 and 14% in part 2). Serious AEs occurred in 12 patients, and there was no treatment-related death. In part 2, most patients had been heavily pretreated: 59% had received more than 5 lines of therapy, 89% were refractory to both IMiD and PI, 37% were refractory to daratumumab, and 89% had received ASCT. ORR was 60% in part 2, with sCR 3%, CR 6%, and VGPR 43%. In the updated report, the median PFS was 12 months and mDOR was 14.3 months. These results suggested that GSK2857916 was well tolerated and induced a good response in patients with RRMM [66]. In the phase II DREAMM-2 study (NCT03525678) [67], 196 patients refractory to PIs, IMiDs or anti-CD38 antibody were recruited and were subsequently stratified by previous lines of therapy (≤ 4 vs. > 4) and cytogenetic risk status to receive the ADC at a dose of either 2.5 mg/kg or 3.4 mg/kg, respectively. To date, the ORR was 31% (30 patient, 97.5% CI, 20.8 to 42.6) in the 2.5 mg/kg cohort and 34% (34 patients, 97.5% CI, 23.9 to 46.0) in the 3.4 mg/kg cohort, and the median OS was not reached. Two deaths were potentially treatment related (one case of sepsis in the 2.5 mg/kg cohort and one of hemophagocytic lymphohistiocytosis in the 3.4 mg/kg cohort). Several trials using GSK2857916 are also underway, combining it with IMiDs (NCT03715478), PIs (NCT03544281) and checkpoint inhibitors (NCT03848845).

Besides BCMA, there are many ADCs currently in development, including those targeting CD56 (lorvotuzumab mertansine), CD38, and CD74 (STRO-001) [68]. In a phase I clinical trial of lorvotuzumab mertansine (NCT00346255) [69], 37 patients were enrolled in the dose escalation phase. The maximum tolerated dose was found to be 112 mg/m^2^, which was subsequently used in the dose expansion phase. AEs were seen in 33 patients, including headache, fatigue, paresthesia, neuropathy and increased transaminase. Disease stabilization was the most frequent response in patients (42.9%). Additionally, 5.7% patients achieved PR and 11.4% patients showed minor response. The patients had an mDOR of 15.5 months and PFS of 6.5 months. To date, all of these ADCs are still being tested in clinical trials.

## Immune checkpoint inhibitors

Cytotoxic T-lymphocyte-associated antigen 4 (CTLA-4)/CD28 and the programmed death-1 (PD-1)/programmed death ligand-1 (PD-L1) are the major immunosuppressive regulatory molecules. CTLA-4 is an inhibitory receptor expressed on T cells and negatively regulates immunity. Currently, ipilimumab, a humanized anti-CTLA-4 monoclonal antibody, has been approved by the FDA for the treatment of a variety of solid tumors, but no clinical trial of ipilimumab for MM has been reported. A phase I clinical trial of ipilimumab for the treatment of recurrent hematological malignancies after stem cell transplantation enrolled a total of 28 patients, including 1 patient with pulmonary plasmacytoma. The patient with plasmacytoma achieved PR and remained progression free for more than 21 months.

The expression of PD-L1 is not only increased in myeloma cells but also in bone marrow stromal cells. In addition, it is markedly elevated in patients with RRMM [70]. A number of pre-clinical and clinical trials targeting the PD-1/PD-L1 axis are currently underway. Nivolumab and pembrolizumab are two of the major PD-1 inhibitors, while durvalumab is a PD-L1 inhibitor. Early clinical data suggested that the inhibitors alone were not effective. The nivolumab phase 1b study in the treatment of RRMM did not observe a treatment response, and likewise, the pembrolizumab phase 1b trial reported that 93% of the patients discontinued treatment due to disease progression [71]. These studies suggested that blocking PD-1/PD-L1 might need to be combined with other treatment strategies.

KEYNOTE-183 is a randomized, open-label, phase 3 trial that uses POM and DEX with or without pembrolizumab to treat patients with RRMM that had previously received at least two lines of therapy [72]. A total of 249 patients were included and randomly assigned to the pembrolizumab group (*n* = 125) and the control group (*n* = 124). With a median follow-up time of 8.1 months, ORR was 34% in the pembrolizumab group versus 40% in the control group and the HR for death was 1.61. Grade 3–5 toxicity increased by 18% in the pembrolizumab group compared to the control group, serious AEs were 63% versus 46%, and treatment terminations caused by AEs were 20% vs. 8%. The immune-related AEs in the pembrolizumab group were mainly pneumonitis, hyperthyroidism, and rash. KEYNOTE-185 evaluated pembrolizumab in combination with Len and DEX in the treatment for patients with NDMM who were ineligible for ASCT. Patients in the pembrolizumab group faced more than double the risk of death than those in the control group. Non-progressive causes of death included pneumonia, intestinal bleeding, and heart failure. In July 2017, the FDA terminated KEYNOTE-183 and185 and multiple other clinical trials using PD-1 and PD-L1 inhibitors in combination with Len and POM due to safety issues (NCT02036502, NCT02726581, and NCT01592370).

Related trials, e.g., on nivolumab, in the treatment of RRMM had also been conducted. However, Checkmate-039 (nivolumab combined with daratumumab, with or without POM and DEX) and Checkmate-602 (nivolumab, elotuzumab, POM and DEX) trials had been terminated by the FDA due to the safety issues of pembrolizumab.

The majority of the current clinical trials focus on the combination of checkpoint inhibitors and IMiDs. However, IMiDs may reduce the expression of PD-1 in T and NK cells as well as the expression of PD-L1 in myeloma cells and myeloid-derived suppressor cells. Therefore, the combination of the two may lead to a decrease in the effector cell targets, which may reduce the corresponding antibody binding. In addition, the downregulation of PD-1/PD-L1 may lead to the compensatory upregulation of other immune checkpoints [73]. Therefore, the use of immune checkpoint inhibitors in the treatment of MM remains challenging. Currently, treatment options using immune checkpoint inhibitors in combination with radiotherapy [74], tumor vaccines [75], chemotherapy and bone marrow transplantation [76], CAR-T [77], and other immune checkpoint inhibitors are being explored.

### Vaccine

A vaccine targeting tumor cells is also an immunotherapy by inducing CD8 + cytotoxic T lymphocyte [78]. The success of vaccination depends on selecting the appropriate patient population, targeting antigens that are selectively expressed on tumor cells, and using a combination of methods to effectively induce and maintain antigen-specific anti-tumor immune responses. Currently, many MM cell antigens are targeted, and vaccines using whole or partial protein sequences are combined with autologous stem cell transplantation and lymphocyte infusion for treatment. It has been speculated that, after allo-SCT or ASCT when the tumor load is low, lymphocyte infusion will help restore immune capacity through the expansion of low levels of Treg and CD8 + cells, and vaccination at this time can trigger tumor antigen-specific immunity [79]. Cohen et al. [80] recruited 13 patients with MM (NCT01380145) to apply autologous lymphocyte infusion and peri-ASCT immunotherapy with MAGE-A3 vaccination. The combined immunotherapy produced high titers in humoral immunity and strong antigen-specific CD4 + T cell responses in all subjects, and the responses could last for at least one year after ASCT. However, this study, like some other studies, did not observe a significantly improved clinical outcome, which was probably attributed to the heterogeneity of MM cells and inability of a single tumor-associated antigen to illicit sufficient immune response to control the disease. A phase II clinical study [81] tested a vaccine derived from the fusion of patient autologous dendritic cells and tumor cells in patients with RRMM after ASCT. By design, this vaccine was expected to stimulate a broad antitumor response. No overt safety issue was observed and MM-specific T cells expanded significantly in patients. 70% of the patients were stable in 2 months and 24% of the patients showed improved treatment response. Currently, a multi-center randomized clinical trial (NCT02728102) is underway to evaluate the efficacy of this combined therapy using vaccine and ASCT.

## Bispecific antibodies and bispecific T-cell engagers

Recently, another new strategy of immunotherapy using bispecific antibodies and bispecific T-cell engagers (BiTEs) has been shown to have more advantages than monospecific antibodies. These agents have two main binding domains: one is binding to the surface antigen of the tumor cells, and the other is for the immune effector cells, such as CD3 of T cells or CD16 of NK cells. These interactions do not depend on T cell receptor (TCR) specificity and antigen presentation but directly leads to the aggregation of the cells involved, activation of effector cells, and killing of the tumor cells [82]. Bispecific antibodies are classified into IgG-like and non-IgG-like types based on the presence or absence of an Fc region. Typically, IgG-like antibodies are more stable than non-IgG-like types. They have a longer half-life and act through antibody-dependent cellular cytotoxicity, antibody-dependent cellular phagocytosis, or complement-dependent cytotoxicity through Fc mediation. BiTEs are non-IgG-like and contain two single variable regions on the structure. They have a small molecular weight and short half-life and require continuous infusion to maintain the therapeutic level of the agent [83].

Currently, blinatumomab, a CD3/CD19-BiTE has been approved by the FDA for relapsed and refractory acute lymphoblastic leukemia [84]. MM cells do not express CD19, but in vitro experiments have found that CD19 + B lymphocytes can promote MM cell clonal expansion and may play the role of myeloma stem cells; hence, it seems to be reasonable to use blinatumomab for MM [85]. However, in order to maximize the therapeutic effect of BiTE, the selected target should be expressed only on tumor cells. The BCMA is under intensive focus. The first BiTE used for MM treatment is BI 836,909, which targets CD3 to activate T cells and targets BCMA to selectively induce BCMA-positive MM cells to lyse. Noticeably, its activity is not affected by bone marrow stromal cells, soluble BCMA, or proliferation-inducing ligands. Moreover, BI 836,909 could prolong the survival time in a mouse xenograft model [86]. It was acquired by AMGEN as AMG 420 for a phase I clinical trial for RRMM (NCT02514239) [87]. A total of 42 patients received continuous intravenous infusion at a dose of 0.2–800 µg/day. Each course consisted of treatment for 4 weeks followed by an interval of 2 weeks with no treatment, with a maximum of 10 courses. The patients had a median age of 65 years and had received an average of 4 lines of therapy. During AMG 420 treatment, the patients averaged 2.5 courses and 50% of them developed serious AEs, mainly infection (*n* = 12) and polyneuropathy (*n* = 2). Three patients developed grade 2 or 3 cytokine release syndrome (CRS), such as confusion and aphasia, similar to that seen in CAR-T cell therapy. Atypical infections were also observed, such as aspergillosis and fulminant hepatitis. Of the 10 patients who received a dose of 400 µg/day, 5 achieved MRD-negative sCR, 1 VGPR, and 1 PR, with a response rate of 70% and a response duration of 5.6–10.4 months. Therefore, 400 µg/day was determined as the dose for subsequent treatment. Due to the inconvenience of continuous infusion and the possibility of increasing the risk of infection with non-IgG-like bispecific antibodies, current studies are inclined to IgG-like type that can be administered once or twice a week; however, the corresponding toxicity may increase [84]. AMG 701 is a half-life extended BiTE targeting BCMA. Related clinical studies on monotherapy (NCT03287908) or in combination with IMiDs were ongoing. Another bispecific antibody, CC-93269, which binds bivalently to BCMA and monovalently to CD3, was also reported to have a promising efficacy and a manageable safety profile [88].

Although different agents have different structures or binding sites, most BiTEs target BCMA and CD3. Some BiTEs (e.g., AFM26) target BCMA and CD16 to induce NK cell-mediated cell lethality [89]. Thorsten et al. reported that a novel trispecific, tetravalent antibody that simultaneously targeted BCMA, CD200, and CD16A (FcγRIIIa) could significantly increase binding activity and induce NK cell-mediated cell killing [90]. The results are currently limited to in vitro and animal model studies.

Based on the known significant effect of CD38 monoclonal antibody on MM cells, targeting CD3 together with CD38 is expected to be a promising strategy. In vivo experiments showed that AMG 424, an anti-CD38/CD3 XmAb T cell-recruiting antibody, promoted tumor cell killing by T cells without causing excessive release of cytokines even if MM cells expressed low CD38 [91]. In addition, in vitro and in vivo experiments showed that an anti-FcRH5/CD3 T cell-dependent bispecific antibody at picogram concentrations could lead to the accumulation of FcRH5 on MM cells to stimulate the formation of immune synapses and exclude the inhibitory CD45 from the immune synapses, which is necessary for TCR triggering and T-cell activation. Currently, a phase I clinical trial (NCT03275103) is underway [92].

Recently, G protein-coupled receptor class C group 5 member D (GPRC5D) was found to be overexpressed in MM cells. Pilarisetti et al. constructed a GPRC5D/CD3 bispecific antibody, JNJ-64407564, which could recruit CD3 + T cells to aggregate on GPRC5D + MM cells for cell lethality [93]. A phase I clinical trial (NCT03399799) evaluating JNJ-64407564 in RRMM is currently in progress.

## Chimeric antigen receptor (CAR)-T cells

CAR-T cell immunotherapy is a new milestone in tumor treatment. Gene editing technology introduced chimeric antigen receptors and costimulatory molecules into T cells. The modified T cells, while have the ability to self-replicate, also carry antigens receptors that specifically recognize tumor cells and execute killing. The selection of the target antigen determines the efficacy of CAR-T cells on MM cells. The choice of antigen is determined by two aspects: (1) whether the targeted antigen is accurately and specifically expressed on the malignant tumor cells. The more accurate and specific the antibody is, the more effective the CAR-T cells are to kill the tumor cells; (2) it should not be expressed in normal cells so that the toxic side effect of CAR-T cells could be prevented. As a clonal malignant tumor, MM could have multiple subclones as the disease progresses. These subclones may have diverse antigens because of their genetic and phenotypic differences. A variety of CAR-Ts against different targets have been used in clinical studies of MM.

### BCMA-targeted CAR-T cell trials

The first clinical trial of BCMA-CAR-T cells was conducted by Ali et al. (NCT02215967) [94]. This CAR-T used gamma-retrovirus as a vector and contained a CD28 costimulatory domain. Twelve patients participated in this dose climbing study. Ten of the twelve patients were treated with CAR-T cells at a low dose (0.3 × 10^6^–3.0 × 10^6^/kg); 1 patient achieved VGPR, 1 patient achieved PR, and the remaining 8 patients had SD. The other 2 patients were treated with high-dose (9.0 × 10^6^/kg); one of them achieved sCR but relapsed quickly, while the other one was in VGPR for a long time after treatment. With the increasing dose of CAR-T cells throughout the treatment, the efficacy improved, although more severe side effects and more symptoms of CRS developed. This trial proved for the first time that, in addition to targeting CD19, CAR-T cells targeting other antigens also had significant efficacy in the treatment of MM. In 2018, data were updated on this study [95]. Sixteen patients with RRMM who had received a median of 9.5 (range 3–19) lines of therapy before enrollment received BCMA CAR-T cell infusions at the highest dose of 9 × 10^6^/kg CAR-T cells. The ORR was 81%, including sCR in 2 patients and VGPR in 8 patients. The median event-free survival (EFS) was 31 weeks. The toxicity was substantial as 6 patients required vasopressors. Patients with a high tumor burden experienced CRS of grade 3 or 4.

Bluebird has developed two CAR-Ts: bb2121 is a second-generation CAR-T containing 4-1BB costimulatory domain, and bb21217 has the phosphoinositide 3-kinase (PI3K) inhibitor bb007 added to bb2121. In 2019, a phase 1 study (NCT 2,658,929) evaluated the efficacy of bb2121 in 33 patients with RRMM who had previously received at least three lines of therapy [96]. During the dose-escalation phase, patients were given a single infusion of CAR-T cells at a dose of 50 × 10^6^, 150 × 10^6^, 450 × 10^6^, or 800 × 10^6^; during the expansion phase, the dose was 150 × 10^6^–450 × 10^6^. The ORR was 85%, including 15 (45%) patients with CR (but 6 of the 15 subsequently relapsed). The PFS was 11.8 months (95% CI 6.2–17.8). Sixteen patients were MRD-negative (≤ 10^–4^ nucleated cells). The most common grade ≥ 3 AEs were hematological toxicities. CRS occurred in 25 (76%) patients (23 had grade 1 or 2, and 2 had grade 3). Fourteen (42%) patients had neurotoxicity; of these, 13 had grade 1 or 2, and 1 had reversible grade 4 neurotoxicity. CAR-T cells could still be detected up to 1 year after infusion and their expansion was noted to be associated with remission.

The preliminary data for the ongoing phase 1 clinical study on bb21217 (CRB-402) was released at the 2019 ASH Annual Meeting [97]. In this study, 22 patients received the CAR-T therapy (12 at a dose of 150 × 10^6^/kg, 6 at 200 × 10^6^/kg, and 4 at 450 × 10^6^/kg). Patients had previously received an average of 7 lines of treatment, 18 patients had ASCT, and 7 patients had high-risk cytogenetics. The median follow-up time was 23 weeks. CRS was present in 13 patients, 5 in grade 1, 7 in grade 2, and 1 in grade 3. Five patients developed neurotoxicity, of which 1 had grade 4 and 4 had grade 3, which improved after treatment. Fifteen (83%) patients achieved treatment response but six subsequently had disease progression. Ten patients were MRD-negative (≤ 10^–5^ nucleated cells) by new generation sequencing after 1 month, 6 out of 8 patients were positive for CAR-T cells after 6 months, and 2 out of 2 patients after 12 months.

Effort has been made constantly in the transformation of CAR-T to improve the efficacy. The LCAR-B38M is different from other BCMA CAR constructs in that, in addition to the 4-1BB co-stimulatory domain, there are two BCMA-targeting single-domains, VHH1 and VHH2, which have strong binding capabilities. The phase I single-arm, open-label, multicenter study LEGEND-2 (NCT03090659) in China included 57 patients with RRMM [98]. Patients had previously received an average of 3 lines of therapy (range 1–9). Single-drug cyclophosphamide was used for lymphodepletion, and then LCAR-B38M CAR-T cells (median 0.5 × 10^6^/kg; range 0.07–2.1 × 10^6^) were infused in 3 infusions (20%, 30%, and 50% of total dose). The ORR was 88%. At a median follow-up of 8 months, 39 (68%) patients achieved CR, 3 VGPR, and 8 PR. Flow cytometry analysis revealed 36 patients with negative MRD. In addition, LCAR-B38M effectively reduced extramedullary masses. The most common (≥ 40%) AEs of any grade included pyrexia (91%), CRS (90%), thrombocytopenia (49%), and leukopenia (47%). Although 90% of patients had CRS, most had grades 1 or 2 (83%) and only 4 patients had grade 3, and 1 patient had neurotoxicity (grade 1 aphasia, agitation and seizure-like activity). The study did not find any significant correlation between BCMA expression levels and clinical response. The ORR was 92% in patients with BCMA expression < 40% and 82% in patients with expression ≥ 40%. In addition, no correlation was established between BCMA expression and median PFS or OS, and between the number of CAR-T cells and the clinical response. At the 2019 ASH Annual Meeting, the updated results indicated that the median follow-up was 19 month and the median OS had not yet been reached [99]. The OS at 18 months was 68% (range 54–79%) with an mDOR of 22 months (range 13–29).

In a study on LCAR-B38M (NCT03090659) [100], 17 patients with RRMM underwent CAR-T cell infusion after lymphodepletion. Eight patients in group A received lymphodepletion regimen of cyclophosphamide plus fludarabine followed by 3 CAR-T cell infusions on days 0, 3, and 6, and 9 patients in group B received lymphodepletion regimen of cyclophosphamide followed by 1 CAR-T infusion on day 0. Overall, the most common AE was CRS, with 10 cases of mild and 6 cases of severe but controllable. One patient died from severe toxicity. The ORR was 88.2%, which included 14 sCR and 2 VGPR. All the 14 patients with sCR exhibited negative MRD by 8-color flow cytometry. This study showed that patients who had undergone ASCT previously displayed a long-lasting response with the CAR-T therapy. Recently updated data indicated that 6 patients remained progression-free after a median follow-up of 22 months [101]. The median PFS was 12 months, and the median PFS for MRD-negative patients with CR was 18 months. The median OS had not yet been reached. The PFS was prolonged in group A as compared with that in group B. Two patients in group A and 8 patients in group B showed disease recurrence or progression.

The above studies indicated that BCMA CAR-T cells that target two sites is a novel option for the treatment of RRMM. Phase Ib/II clinical trials are currently underway in the USA (CARTITUDE-1, NCT03548207, and JNJ-68284528), and phase 2 confirmatory studies are being conducted in China (CARTIFAN-1 and NCT03758417). All the above studies were based on lymphodepletion with cyclophosphamide and fludarabine and a single infusion of CAR-T cells. In the CARTITUDE-1 Phase Ib study [102], 29 patients with RRMM who had previously experienced an average of 5 lines of therapy (range 3–18) received a median dose of 0.7 × 10^6^/kg CAR-T cells. The results showed a 100% ORR, including 97% VGPR or better (86% sCR) and 3% PR. The 9-month PFS was 86% and 22 of 29 patients remained alive and progression free at the time of data cut-off. Evaluable patients (*n* = 16, 81%)) achieved MRD negativity (at 10^–5^ or 10^–6^ nucleated cells) at the time of suspected CR.

Currently, there are many ongoing CAR-T studies for BCMA, such as JCARH125 [103], CT053 [104], MCARH171 [105], and CT103A [106]. These studies also showed a good response rate, and no new treatment toxicity and side effect was observed. Key CAR-T clinical trials targeting BCMA are summarized in Table 3.

### CD19-targeted CAR-T cell trials

Although CD19 is not considered as an effective target for MM immunotherapy, some reports demonstrated that a small number of MM cells express B-cell phenotype [107]. Alfred et al. combined a CTL019 CAR-T cell infusion with melphalan and ASCT in a patient with RRMM who had been treated with ASCT 4 years prior but only achieved a short PR [108]. CTL019 CAR-T cells contain CD3-zeta/CD137-based anti-CD19 chimeric antigen receptor from a lentiviral vector. Surprisingly, sustained complete remission was obtained in this patient. Based on this report, a subsequent clinical study (NCT02135406) was carried out [109]. Ten patients who had previously received ASCT but relapsed within 1 year were given melphalan and ASCT, followed by CTL019. This study showed that ASCT combined with CTL019 was safe and effective, with 1 case of sCR, 1 case of VGPR, 2 cases of PR, and the remaining 6 cases had no disease progression. The peak frequency of CTL019 cells in the bone marrow and the presence of humoral and cellular immune responses to the stem cell antigen Sox2, a transcription factor that governs self-renewal and pluripotency with myeloma-propagating capability, were correlated with prognosis [110]. These observations indicated that CTL019 might target CD19 + myeloma-propagating cells.

### CD138-targeted CAR-T cell trials

CD138 is highly expressed on MM cells. Guo et al. [111] reported a CD138-CAR-T with an increasing dose phase I clinical trial (NCT01886976) for the treatment of RRMM. Four out of five patients had SD, and CAR-T cells were still detectable 3 months after treatment. All patients well tolerated the treatment without severe toxicity. A phase I CAR-T trial targeting CD138 is currently being conducted (NCT03672318).

### CD38-targeted CAR-T cell trials

CAR-T targeting CD38 is also under development. These CAR-T cells are associated with AEs, because CD38 is widely expressed in a variety of cell types, including plasma cells, precursor B cells, T cells, NK cells, and myeloid precursor cells, as well as in various organs, such as the gastrointestinal tract and prostate. Attacking CD38-positive normal hematopoietic stem cells and monocytes by second-generation retroviral CD38-CAR-T cells causes severe bone marrow suppression [112]. Drent et al. demonstrated that CAR-T cells could specifically attack CD38-positive MM cells by changing the structure of the light chain and optimizing the affinity of the single-chain variable fragment [113].

### SLAMF7-targeted CAR-T cell trials

SLAMF7 is expressed in > 90% of MM cells. SLAMF7 CAR-T cells derived from the huLuc63 antibody (elotuzumab) have been shown to kill not only the bone marrow MM cells but also extramedullary MM cells in murine xenograft model [114]. Unlike myeloma-specific antigens, SLAM7 is also expressed on immune cells, and the CAR-T cells can selectively effectuate fratricide on SLAMF7 (+/high) NK cells, CD4 (+) and CD8 (+) T cells, and B cells. In addition, the CAR-T cells spare the SLAMF7 (-/low) fraction in each cell subset and preserve the functional lymphocytes. Currently, a couple phase I clinical studies (NCT03710421 and NCT03778346) are underway.

### Light chain-targeted CAR-T cell trials

CAR-T cells designed to act only on the kappa (*κ*) light chain are intended to kill the *κ* chain-expressing neoplastic plasma cells while preserve the lambda chain-expressing normal plasma cells to reduce the immunosuppression by the CAR-T cells. Of the 7 patients included in a phase I clinical trial, 4 were in stable condition and no significant effect was observed in the remaining subjects. The CAR-T cells did not induce serious AEs and were tolerable. It is likely that the low expression level of the *κ* light chain on the surface of MM cells has impaired treatment efficacy [115].

### Problems and solutions in CAR-T cell therapy

Among a variety of AEs in CAR-T cell therapy, CRS is the most frequent and prominent reaction. CRS is a syndrome caused by a large, rapid release of inflammatory factors during CAR-T therapy, leading to a series of clinical manifestations. Due to the difference in CAR-T targets, the occurrence time and intensity of CRS are different. Typically, the degree of CRS in patients with MM is relatively light, and the incidence of grade 3 or 4 is low. Once the symptoms of severe CRS appear, the IL-6 receptor antagonist tocilizumab or steroids should be used as early as possible to reduce the damage of CRS on organ function. Researchers speculated that CAR-T cell toxicity is related to the synthetic nature of the receptor design. Therefore, a new type of CAR-T cells has been designed with an MHC-independent receptor T cell antigen coupler, which can co-opt the endogenous TCR and exert antitumor effect with fewer toxic reactions [116]. Other AEs include persistent cytopenia, hypogammaglobulinemia, and inflammation; all these could be managed through appropriate supportive treatments but need close monitoring.

Although the causes of MM relapse after CAR-T cell therapy are not well known, antigen escape is considered as one of them. Multiple studies have confirmed that tumor cells can downregulate target antigens and tumor cell clones with expression of epitope different from CAR-T targets may emerge after a period of time [117, 118]. CAR-T cells could activate trogocytosis and transfer the target antigens to T cells, thereby reducing the concentration of the target antigen on tumor cells and leading to the self-killing and depletion of T cells [119]. In order to overcome antigen loss or epitope change and improve efficacy, targeting multiple antigens is a good treatment approach, including injection of CAR-T cells designed by two different strategies or bispecific CAR-T cells possessing two complete and independent CARs [120, 121]. A single-arm phase 2 study in China evaluated the clinical efficacy of mixed injections of anti-CD19 and anti-BCMA CAR-T cells in the treatment of RRMM. Twenty out of twenty-one (95%) patients exhibited treatment response, including 9 sCR, 3 CR, 5 VGPR, and 3 PR. The major AEs were grade 1 or 2 CRS with no treatment-related death [121]. At the 2019 ASH Annual Meeting, a clinical study of dual-target BM38 CAR-T for RRMM was reported [119]. The BM38 CAR contains the anti-CD38 and anti-BCMA single-chain variable fragment in tandem plus 4-1BB signaling and CD3 zeta domains. Ten of sixteen (62.5%) patients had genetic abnormalities and 5 (31.25%) had extramedullary lesions. Fourteen (87.5%) patients achieved ORR, 8 (50%) sCR, 2 (12.5%) VGPR, and 4 (25.00%) PR, and 14 (87.5%) showed negative MRD in bone marrow. The longest duration of sCR was > 51 weeks, and 5 (62.5%) of the 8 patients had still maintained the sCR. Intriguingly, the extramedullary lesions of the 5 patients disappeared completely. No overt neurotoxicity was observed, while CRS and other toxicity could be controlled. The above studies indicated that CAR-T cell therapy targeting multiple antigens exhibits a high response, which may significantly improve therapy in RRMM.

In addition to antigen escape, the inability of CAR-T cells to survive in the body for a prolonged period is also one of the causes of relapse. Thus, investigators seek to increase the proportion of memory T cells through various editing and construction techniques to achieve long-term survival of CAR-T cells. The bb21217 contains a PI3K inhibitor structure, which increases the proportion of memory-like T cells. Previous studies have also suggested that adjusting the ratio of CD4/CD8 could improve the efficacy of CAR-T cells. A study of BCMA CAR-T cells with an equal number of CD4 + and CD8 + T cells reported that 7 patients displayed a response to treatment after 28 days of infusion, with a median survival of 16 weeks [122]. In addition, a new CAR-T, P-BCMA-101 containing CARtyrin, showed a good therapeutic response and safety in the treatment of RRMM [123]. The CAR-T cells were mainly stem cell-like memory T cells, which had a long life span, self-renewal capacity, and pluripotency. Moreover, the duration of treatment response was also increased. Furthermore, the use of the non-viral piggyBac transposon-based delivery system in CAR-T cells may reduce the risk associated with viral vectors [124].

To further improve the efficacy, clinical research on the combination of CAR-T cells and drugs is being conducted. For example, Len could enhance the therapeutic effect of CS1 CAR-T cells [125]. However, the potential AEs of these drugs have not yet been fully revealed and the combination of multi-target drugs might further increase the incidence of AEs.

Producing specific CAR-T cells is complicated and expensive, and exploring “universal” CAR-T cell technology is a promising research direction. Universal CAR-T cells derived from healthy donors have the potential to overcome immune deficiencies associated with tumor therapy. In addition, universal CAR-T cell products can streamline the engineered cell manufacturing processes and allow for large-scale production. Compared to the patients' autologous specific T cells, universal CAR-T cells might yield fast and cost-effective results. In April 2019, the FDA approved UCARTCS1 as the first allogeneic CAR-T cell therapy for MM. UARTCS1 is based on a tailored manufacturing process developed by Cellectis, which removes both the CS1 antigen and TCR from the T-cell surface using TALEN® gene-editing technology before adding the CS1 CAR construct. This method includes lymphodepletion and CAR-T cell cross-reaction. However, the principle and efficacy of the universal CAR-T cells require further validation.

Currently, CAR-T is an emerging treatment at its exploratory stage. The therapy often bridges with hematopoietic stem cell transplantation. It is intriguing to see whether it could be part of the overall strategy in the treatment of MM.

## Alkylating agents

### Bendamustine

Bendamustine is a dual-functional nitrogen mustard derivative that acts as an alkylating agent and inhibits metabolism. The phase III clinical trial conducted by the East German Study Group of Hematology and Oncology found that, compared with the melphalan and prednisone regimen (MP), a combination of bendamustine and prednisone (BP) could significantly increase the response rate in patients with NDMM and improve the survival [126]. The BP regimen was approved in Europe in 2010 as first-line regimen for the treatment of ASCT-ineligible patients more than 65 years old with neuropathy induced by the treatment of BTZ and/or thalidomide [127].

Previous studies mainly used bendamustine in combination with other drugs (glucocorticoids, PIs, or IMiDs) as an alternative for patients with RRMM [128]. The triplet of bendamustine, ixazomib, and DEX (BID) was used in a phase I/II trial in patients with RRMM who previously had received a median of 4 (range 4–9) lines of therapy. With a median follow-up of 17 months, 11% achieved VGPR and 50% achieved partial response, with median PFS of 5.2 months (95% CI 1.96–8.3) and OS of 23.2 months (95% CI 16.3–30.07) [129]. Another phase II study evaluated the bendamustine, BTZ and DEX regimen (BBD) as a front line therapy for patients who were not candidates for high dose chemotherapy [130]. It is worth noting that patients received 2 different sets of doses and cycles in this trial, the original and the modified. The original regimen was efficacious but relatively toxic in early analysis, so the regimen was amended as: bendamustine 80 mg/m^2^, days 1, 2; BTZ 1.3 mg/m^2^, days 1, 8, 15; DEX 20 mg, days 1, 2, 8, 9, 15, 16, every 28 days up to 8 cycles, then maintenance therapy with 1.3 mg/m^2^ iv BTZ every 2 weeks. The ORR was 91% and CR was 9% in the 59 patients enrolled. With a median follow-up of 19.1 months, the median PFS was 11.1 months and 18.9 months for the original and modified regimens, respectively. The most common grade ≥ 3 AEs were fatigue and neuropathy.

## Bcl-2 inhibitors

### Venetoclax (ABT199)

Tumor cells harboring t(11;14) were associated with high expression of Bcl-2. Overexpression of anti-apoptotic proteins (e.g., Bcl-2 and Bcl-XL) could favor the survival and drug resistance of tumor cells [131]. A phase Ib clinical trial (NCT01794507) found that the ORR of non-BTZ-resistant patients (*n* = 66) was 67% when treated with venetoclax (VEN) in combination with BTZ and DEX. The ORR reached 82% in non-IMiDs-resistant patients and 57% in IMiDs-resistant patients. In PI resistant patients, the ORR still reached 32% (*n* = 28) [132]. A retrospective analysis of the BELLINI (NCT02755597) study found that patients with high expression of cyclin D1 due to t(11;14) had better response to the combination of VEN, POM, and DEX [133]. The phase II STORM study (NCT02336815) showed that for patients resistant to BTZ, carfilzomib, Len, or POM, treatment with VEN and DEX could still achieve an ORR of 21% [134]. Another phase II clinical trial (NCT02899052) evaluated the effect of the carfilzomib and DEX plus VEN regimen (VenKd) in RRMM. Among 42 patients studied, 93% had been previously exposed to PIs (with 50% being refractory), 62% had not responded to IMiDs, and 33% had been PI and IMiDs dual-refractory. The results showed that ORR was 78% and VGPR was 56%. The ORR in patients with t(11;14) translocation was even higher (close to 100%) [135]. A multi-center, randomized, open-label phase III study (NCT03539744) is currently on going and is focused on the efficacy and safety of VEN plus DEX versus POM plus DEX regimens in patients with t(11;14) positive RRMM.

## XPO-1 inhibitors

### Selinexor

Exportin-1 (XPO-1) is in charge of the export of proteins from the nucleus. Previous studies [136] showed that XPO-1 inhibitors had high anti-MM effect in monotherapy or in combination with PIs, IMiDs, anthracycline, or alkylating agents [134, 137]. The phase IIb STORM trial (NCT02336815) [134] included patients with RRMM resistant to multi-drugs (83 out of 123 patients resistant to BTZ, carfilzomib, Len, POM, and daratumumab). The median number of previous lines of therapy was seven and 53% of the patients had high-risk cytogenetic abnormalities. The results showed that patients receiving selinexor plus DEX achieved a partial or better response rate of 26% (95% confidence, interval 19–35). The mDOR was 4.4 months, median PFS was 3.7 months, and median OS was 8.6 months. Previous results of STOMP (NCT02343042) showed that the ORR of selinexor combined with BTZ and DEX was 63% (84% ORR for PI non-refractory and 43% for PI refractory patients), and the duration of the response was prolonged. The median PFS was 9.0 months (17.8 months for PI non-refractory and 6.1 months for PI refractory patients). Grade 3 or 4 myelosuppression was the main AEs. In addition, combination with BTZ could reduce the side effect of selinexor on the gastrointestinal tract. The ongoing phase III BOSTON trial (NCT03110562) aimed to evaluate the efficacy of selinexor in combination with BTZ and DEX (SVD) in patients with RRMM who had failed in one to three lines of therapy. Current data showed that the median PFS of the SVD group was extended by 4.47 months compared with the VD (13.93 months vs. 9.46 months, *p* = 0.0066) and the ORR in SVD group was superior to that in VD group as well (76.4% vs. 26.3%, *p* = *0*.*0012*). In 2019, the US FDA approved the regimen of selinexor plus DEX for treating patients with RRMM who had not responded to at least four treatments (including two PIs, two IMiDs, and a monoclonal anti-CD38 antibody).

## Kinesin spindle protein inhibitors

### Filanesib (ARRY-520)

Filanesib, also known as ARRY-520, a kinesin spindle protein (KSP) inhibitor, could block the separation of centrosomes and assembly of spindles in the early stage of cell mitosis, and thus inhibit cell proliferation. As KSP is essential to the cells undergoing cell division, it has been considered as a potential target for the treatment of cancers [138]. Various KSP inhibitors were shown to have strong anti-tumor effect in vitro. Neutropenia and mucosal inflammation were the most common dose-limiting toxicities as shown in a phase I trail (NCT00821249). In phase II of this trial, grade 3 and 4 cytopenia were reported in approximately 50% of patients. Response rates (partial response or better) were 16% for single agent and 15% for filanesib plus DEX in patients with RRMM [139]. In addition, the combined therapy could increase the sensitivity of MM cells to PIs and IMiDs [140, 141]. Patients with a low serum level of alpha 1-acid glycoprotein could better benefit from filanesib treatment [138].

### Melflufen

Melflufen is a peptide hinge alkylation agent that increases the concentration of melphalan derivatives in myeloma cells and other cancer cells that overexpress aminopeptidase. HORIZON (NCT 02963493) is a single-arm, open-label, phase II study to evaluate the effect and safety of melflufen plus DEX in patients with RRMM [142]. The enrolled patients had received IMiDs and PIs as front line therapies and had a poor response to POM and/or daratumumab. Most of the patients had high-risk cytogenetics and medullary lesions. As of February 2019, 95 patients were enrolled and median number of prior lines of therapy was 5 (2–13). 61% of the patients with available cytogenetic data (*n* = 66) had high-risk cytogenetics. ORR was 30% in 90 patients with VGPR 11% and PR 18%. Clinical benefit rate was 40%. Median PFS for all patients treated (*n* = 95) was 4 months (95% CI 3.3–4.7), median OS was 10 months (95% CI 8.1-not reached), and mDOR (*n* = 27) was 4.8 months (95% CI 3.6-not reached). Updated data from EHA 2020 Annual Meeting Abstract [143] reported the time to next treatment (TTNT) in patients with advanced RRMM after melflufen plus DEX in HORIZON study. As of the cutoff date (October 1, 2019), 154 patients had received the treatment and 125 of them were evaluated for TTNT. With a median follow-up of 15.3 months, the median TTNT was 8.0 months (95% CI 7.2–8.9) and the median PFS was 4.2 months (95% CI 3.7–4.9). Similar median TTNT was found in patients with triple-class refractory MM (*n* = 93, 8.1 months, 95% CI 7.1–10.9) and extramedullary disease (*n* = 42, 7.7 months, 95% CI 6.3-not evaluable). The median PFS was almost the same in these two groups (4.0 months, 95% CI 3.0–4.5 vs. 3.0 months, 95% CI 2.1–4.0). More studies are expected to explore the use of melflufen in combination with other therapies as a first-line treatment or in transplant-ineligible patients. The phase I/II O-12-M1 (NCT01897714) [144] study aimed to determine the maximum tolerated dose of melflufen in patients with RRMM and to investigate the safety and efficacy of the combination of melflufen and DEX. In the phase 1 of this trial, the maximum tolerated dose was 40 mg of melflufen on day 1 in 21-day cycles in combination with DEX. No dose-limiting toxicity was observed in three dose groups (15 mg, 25 mg, and 40 mg). In the phase 2 of this trial, patients who had received the combination therapy achieved an ORR of 31% (95% CI 18–47) and clinical benefit rate of 49% (22 of 45; 95% CI 34–64).

### RAS/RAF/MEK/ERK pathway

The RAS/RAF/MEK/ERK pathway regulates gene expression, cell survival, proliferation, migration, and angiogenesis. KRAS/NRAS/BRAF mutations can be detected in up to 50% of patients with MM and 45–81% of patients with RRMM [145]. RAS mutations are associated with disease progression and reduced survival. The t(4;14) translocation can lead to increased FGFR3 expression and stimulate the RAS/MAPK pathway. Cobimetinib, a MEK1 inhibitor, has been approved in combination with vemurafenib, a BRAF inhibitor, for the treatment of melanoma with BRAF V600E or V600K mutation. Based on melanoma studies, a patient with RRMM who had BRAF V600E mutation was treated with cobimetinib plus vemurafenib and achieved a rapid and lasting response [146].

## Conclusions

With increasing understanding of the mechanisms underlying MM in recent years, novel agents and combinations of existing treatment modalities are providing innovative therapies. In particular, immunotherapies including checkpoint inhibitors, vaccine, BiTEs, and CAR-T cells demonstrated promising efficacy in inducing deep and durable remissions in both NDMM and RRMM. As more novel agents make their way through clinical trials, the efficacy of immunotherapies will need to be assessed in different disease subgroups. It also needs to be clarified whether prior treatment with one immunotherapeutic agent influences the efficacy of subsequent lines of therapy. Cell-based immune therapies may serve as the preferred options for patients with high-risk cytogenetics who are more resistant to standard biological therapies. For these patients, treatment with immune therapies in early disease course may provide significant additional benefits. Nevertheless, high-risk cytogenetics associated with high levels of clonal diversity and cell proliferation may render escape mechanisms, such as loss of antigen expression, increased number of immunomodulatory cells in the microenvironment and upregulation of negative costimulatory molecules. Therefore, the suppressive immune impairments and the mechanisms of resistance to immunotherapies need further exploration. With more approved options, potential synergies among immunotherapeutic agents should be evaluated. For example, BiTE may enhance cytotoxic T cell activity followed by cancer vaccination or CAR-T cell therapy. Future study may come to the treatment algorithms of how to make optimal sequence of treatments and how to choose best treatment when disease progresses, while taking into account prior treatments, toxicities, and patient comorbidities and preferences. Although MM currently remains incurable, the future is bright.

## Data Availability

Data sharing is not applicable to this article as no datasets were generated or analyzed during the current study.

## Abbreviations

ADC: Antibody–drug conjugate
AE: Adverse event
ASCT: Autologous stem cell transplantation
BCMA: B-cell maturation antigen
BiTEs: Bispecific T-cell engagers
BTZ: Bortezomib
CAR-T: Chimeric antigen receptor T cells
CTLA4: Cytotoxic T-lymphocyte-associated antigen 4
CR: Complete remission
CRS: Cytokine release syndrome
DEX: Dexamethasone
EFS: Event-free survival
EHA: European Hematology Association
GPRC5D: G protein-coupled receptor class C group 5 member D
HDAC: Histone deacetylase
HDACi: HDAC inhibitor
HDT: High-dose chemotherapy
IMiDs: Immunomodulator drugs
KSP: Kinesin spindle protein
Len: Lenalidomide
mDOR: Median duration of response
MM: Multiple myeloma
MRD: Minimal residual diseases
NDMM: Newly diagnosed multiple myeloma
OS: Overall survival
ORR: Overall response rate
PD-1: Programmed death-1
PD-L1: Programmed death ligand-1
PFS: Progression-free survival
PIs: Proteasome inhibitors
POM: Pomalidomide
PR: Partial response
RRMM: Relapse and refractory multiple myeloma
SD: Stable disease
TCR: T cell receptor
TTP: Time to progression
VGPR: Very good partial response
